# Patulin in Apples and Apple-Based Food Products: The Burdens and the Mitigation Strategies

**DOI:** 10.3390/toxins10110475

**Published:** 2018-11-15

**Authors:** Lei Zhong, Jason Carere, Zhaoxin Lu, Fengxia Lu, Ting Zhou

**Affiliations:** 1 College of Food Science and Technology, Nanjing Agricultural University, 1 Weigang, Xuanwu District, Nanjing 210095, China; 2014208012@njau.edu.cn (L.Z.); fmb@njau.edu.cn (Z.L.); 2 Guelph Research and Development Centre, Agriculture and Agri-Food Canada, 93 Stone Road West, Guelph, ON N1G 5C9, Canada; jasoncarere@gmail.com

**Keywords:** apples, apple-based products, patulin, blue mold, *Penicillium expansum*, detoxification

## Abstract

Apples and apple-based products are among the most popular foods around the world for their delightful flavors and health benefits. However, the commonly found mold, *Penicillium expansum* invades wounded apples, causing the blue mold decay and ensuing the production of patulin, a mycotoxin that negatively affects human health. Patulin contamination in apple products has been a worldwide problem without a satisfactory solution yet. A comprehensive understanding of the factors and challenges associated with patulin accumulation in apples is essential for finding such a solution. This review will discuss the effects of the pathogenicity of *Penicillium* species, quality traits of apple cultivars, and environmental conditions on the severity of apple blue mold and patulin contamination. Moreover, beyond the complicated interactions of the three aforementioned factors, patulin control is also challenged by the lack of reliable detection methods in food matrices, as well as unclear degradation mechanisms and limited knowledge about the toxicities of the metabolites resulting from the degradations. As apple-based products are mainly produced with stored apples, pre- and post-harvest strategies are equally important for patulin mitigation. Before storage, disease-resistance breeding, orchard-management, and elicitor(s) application help control the patulin level by improving the storage qualities of apples and lowering fruit rot severity. From storage to processing, patulin mitigation strategies could benefit from the optimization of apple storage conditions, the elimination of rotten apples, and the safe and effective detoxification or biodegradation of patulin.

## 1. Introduction

The apple fruit (*Malus* sp., Rosaceae) is a nutrient-dense food, which is highly recommended to be included in healthy diets. With various phytochemicals and dietary fiber, apples contribute many health benefits to consumers, and participate in reducing chronic diseases [1,2]. According to the Food and Agriculture Organization (FAO), apples and apple-products (juices, purees, ciders, concentrates, and compotes) placed 17th in the list of the highest produced commodities worldwide [3]. The durability of a seasonal fruit, like apple, is largely affected by postharvest diseases. Approximately 25% and 50% of fruit product losses are caused by fungal pathogens during long term storage in developed countries and developing countries, respectively [4]. In terms of apples, the most common postharvest pathogen is *Penicillium expansum*, which may jeopardize the profitability of apple producers and negatively affect human health because of the blue mold decay and the consequent production of the toxic metabolite, patulin.

Patulin is viewed as a natural contaminant in apple-based products, particularly apple juice and unfermented apple cider. Initially identified as a broad-spectrum antimicrobial agent, patulin was reclassified as a mycotoxin in the 1960s because of its acute toxicity in human beings [5]. The amount of patulin in apple products was then considered as a measure of quality in regard to food safety standards/practices around the world. A provisional maximum tolerable daily intake of patulin has been set at 0.4 µg/kg body weight/day by the Joint FAO/WHO Expert Committee on Food Additives since 1995 [6]. Based on this, the maximum level of patulin has been restricted no more than 50 µg/L in apple juice and apple cider by the European Commission [7], the United States Food and Drug Administration [8], the Ministry of Health of the People’s Republic of China [9], and Health Canada [10]. Solid apple products (e.g., apple sauce) and products for infants and young children have to meet tighter legislations, 25 µg/kg and 10 µg/kg, respectively, as established by the European Commission [7]. The incidence of patulin contamination are a worldwide problem (Table 1), especially for the main producers of apples and apple-based products, such as China, the EU, and the USA [11]. An analysis of 35 apple products from Northeast China showed that 20% of these products have exceeded the current regulatory limit (50 µg/kg), the highest level of which has 94.7 µg/kg patulin in apple juice concentrate [12]. Another survey carried out analyzing 1987 apple juice concentrate samples from Shaanxi (2006–2010) indicated that 97.7% of the products were contaminated by patulin, ranging from 20.0 to 78.0 µg/L [13]. In Poland, 5 µg/L or more patulin was detected in 22% of the 754 apple juice samples collected during the 1993–2003 period [14]. In Italy, a report released in 2005 showed that the amount of patulin was 4.77 µg/L and 10.92 µg/L in conventional and organic apple juices, respectively [15]. In Michigan, USA, it was reported that 23% of apple-juice and cider samples from local groceries were contaminated by patulin, with concentrations ranging from 8.8 to 2700.4 µg/L [16]. Therefore, it is of great importance to investigate the mechanism(s) and causes of patulin incidence in apple products.

Frequently found in the rotten tissues of apples, patulin accumulation correlates with apparent symptoms of blue mold. Apple blue mold usually starts with the invasion of *P. expansum* spores on the wounds of fresh apples. Such stem punctures, insect injuries, and bruises are created during the picking and handling operations in the apple orchard, until the final processing steps of products [30]. For over-ripening apples or apples that were held in storage for too long, open lenticels on the skin are also susceptible and can be easily attacked by the pathogen [31]. Furthermore, the colonization and germination of psychrophilic fungi, such as *P. expansum*, is barely affected by the lower temperature encountered in commercial storage rooms/facilities [32]. As lower-grade or unfit apples are usually used for juice or cider processing, the removal of rotten or damaged apples prior to pressing is strongly recommended by the FAO in order to reduce the occurrence of patulin in the final products [33]. However, even when decayed fruits are diligently culled, patulin has been constantly detected in apple products made from externally healthy apples that have internal rot which is not omitted before pressing. This invisible decay comes from the colonization of *P. expansum* in the opening calyx tube during apple blooming [30,34]. Once patulin is produced, conventional pasteurization only marginally reduces patulin in bottled apple juice [35]. In such cases, the development of strategies to effectively control patulin contamination in apple products during processing is of great importance.

Given the large consumption of apple products among infants and young children, the presence of patulin in apple-based products triggers concerns of food safety in the public. Moreover, the conventional control of blue mold decay by fungicides also concerns the public by itself, because of the potential risks of such chemicals to human health. In the view of blue mold and patulin, studies from the past decade have reviewed patulin-producing strains and their biosynthesis genes [36,37]. Considering that patulin is often detected in apples products, factors that affect patulin production in apple fruits, conventional practices for postharvest control, and the fate of patulin during juice processing have been discussed [32,38,39,40]. In fact, the severity of blue mold is directly associated with the amount of patulin produced in apples, and the later further makes *P. expansum* more aggressive during long time storage [41]. Practices like pasteurization can only eliminate the existence of the pathogen, but not the presence of patulin [35]. Therefore, this review will discuss the factors and challenges that affect patulin production and its removal from apples, as well as the emerging strategies in reducing pathogen infection and patulin contamination in apples from orchards to dining tables, with a focus on the work published in the last decade, with an aim of highlighting future prospective. 

## 2. The Burdens of Patulin Accumulation in Apples and Apple Products

The high level of patulin contamination in apple products continues to be a problem because of the complicated factors that are associated with patulin production, as well as the challenges that hamper the control of patulin levels in apple products. 

### 2.1. Factors Affecting Patulin Production in Apples

A sufficient pathogen load, susceptible apple cultivars, and favorable environments are the fundamental elements required for the occurrence of any plant disease [42]. Widely spread in storage rooms, *P. expansum* is the most important causative agent of blue mold decay and patulin producer in apples. Among the thousands of apple cultivars, some of them show a fast response toward wounding and decaying, while other susceptible ones fail to combat pathogen attack, resulting in a large accumulation of patulin within the fruit’s flesh. 

#### 2.1.1. Toxigenic *Penicillium* Species

Patulin production is often detected within the decayed area, which is majorly caused, by *P. expansum* and other pathogenic *Penicillium*. In a recent study, among the 166 fungal isolates that have been reported to produce patulin, 77 belong to pathogenic *Penicillium* [37]. As a psychrophilic fungus, *P. expansum* grows well at 0 °C or even −2/−3 °C, so infection can still take place during the cold storage of apples [43]. Moreover, the population of *Penicillium* is usually higher within storage facilities than that in the fields. In a French apple orchard, the density of *Penicillium* was less than 50 spores/m^3^, however, 2500 spores/m^3^ of *Penicillium* were detected in the atmosphere of previously disinfected warehouses, where *P. expansum* and *Penicillium solitum* accounted for 20.0–65.6% and 6.0–47.1%, respectively [31]. An isolation of the fungal species in long-term stored “Jonagold” apples in Belgium also indicated that *P. expansum* and *P. solitum* were the dominating species [44]. Although *P. solitum* is unable to produce patulin, it acts as a predisposing agent, which allows the entry of *P. expansum* and causes more destructive infections [37,45]. Moreover, the average population of *Penicillium* on the surface of stored apples was 10–50 spores/cm^2^ in the first month, and went up to 300–400 spores/cm^2^ after the sixth month [31]. While sound apples are unlikely to become infected by the pathogen [46,47], the apples that are exposed to high concentration of *Penicillium* spores could develop severe decay after long-term storage.

Patulin accumulation in apples is affected by the severity of blue mold and the toxin-producing capacity of *P. expansum*. Generally, the severity of the rotten tissue is positively correlated with the patulin content in apples [48]. However, the role of accumulated patulin in the aggravation of blue mold decay in apples has not been fully elucidated in the existing literature yet [49]. A wide range of *P. expansum* strains have recently been reported to produce patulin with a production capacity that is varies from strain to strain. The highest production level of patulin by *P. expansum* isolates from Vineland, Woodstock, and Georgian Bay in Ontario, Canada, was around 0.66 mg/kg [50]. While the *P. expansum* isolate FC116 from Belgian “Jonagold” apple orchards was able to produce 747 mg/kg patulin in vitro [44]. More intriguing, there was no correlation between patulin accumulation in actual apples and in the potato dextrose agar, even by the same *P. expansum* isolate [51]. In addition to the noticed differences at the genetic level between patulin producing *P. expansum* strains, apple cultivars and environmental conditions also play important roles in disease severity and patulin accumulation. 

#### 2.1.2. Traits of Apple Fruits

In nature, patulin is more likely found in apples and apple products, because the physicochemical properties of apple fruits are suitable for *P. expansum* growth. With water activity (*a*_w_) at 0.98–0.99, apple fruits meet the minimal level of *a*_w_ for *P. expansum* spore germination and patulin biosynthesis, which are around 0.83–0.85 and 0.99, respectively [52]. The pH range of the apple (=3.1–4.2) also satisfies *P. expansum* colonization and patulin production (pH 2.5–6) [53,54]. Other than these two factors, different genetic backgrounds and growth conditions shape the physical and chemical traits of apple fruits, which eventually determine the wound healing ability, as well as the susceptibility to blue mold and patulin production in wild or cultivated apples.

The defense system of injured apples, whether resistant or susceptible to *P. expansum* attacks, is highly regulated by specific genetic information. Apple cultivars that share close genetic profiles show a similar response to pathogen attack. When inoculated with same amount of *P. expansum* spore suspension, a similar growth pattern of *P. expansum* was observed on “Golden Delicious” and its descendants “Pink Lady” and “Ariane” apples [55]. Collected from Central Asia (mainly Kazakhstan), the accessions of a wild apple cultivar *Malus sieversii* showed resistant behavior to *P. expansum* infection, and most of the studied cultivars were able to survive the inoculation of *P. expansum* at 1 × 10^3^~1 × 10^4^ spores/mL [56]. The immune accession GMAL 4317.f can even survive the inoculation at 10^5^ spores/mL [57]. To resistant apples, the better basal defense and faster stress response are the result of differential genes expression, including the genes that encode wound-healing or defense-related proteins, as well as reactive oxygen species (ROS) detoxifying enzymes in response to patulin [58]. The latest identification of the qM-*Pe*3.1 quantitative trait loci (QTL) in *M. sieversii* attributes the fundamental reason of susceptible apples is the lack of a peculiar allele that controls blue mold resistance [59]. Beyond the intrinsically genetic strength, the transcriptomic analysis also revealed that the resistant *M. sieversii* had a much more rapid wound-healing behavior than that in the susceptible “Royal Gala” [60]. A similar pattern has also been used to explain the difference of decay incidences among immune, resistant, and moderately resistant *M. sieversii* accessions [61]. It is unfortunate that the patulin content was not evaluated in the abovementioned research. It is most likely that a reduced decay area would in turn translate to reduced levels of patulin in the fruit, even though the patulin accumulation may not be positively correlated with disease severity in all of the studied cases.

Flesh firmness is an important sensory trait of all apple cultivars, particularly domesticated ones, that is associated with the fruit’s resistance to blue mold decay and patulin production. With fruit ripening, the flesh texture becomes softer, which leads to higher risks of pathogen infection [62]. Generally, a negative correlation between the firmness and severity of blue mold in apples has been reported widely. Caused by *P. expansum*, the decay incidences were incredibly high in over-ripe “Golden Smoothee” apples, because of decreased flesh firmness [63]. A similar phenomenon has also been discovered in a total of 28 apple genotypes from Mexico, where the commercially ripe apples showed less susceptibility to *P. expansum* than the over-ripe apples [64]. With poor firmness, rots were developed in over-ripe apples even by non-host pathogen *Penicillium digitatum* [63]. However, the relationship of fruit firmness and disease tolerance differs among early- and late-ripening cultivars. After storage for 6–12 weeks, a negative correlation between the fruit firmness with the lesion diameter of blue mold decay was only found in the late-ripening, but not in the early-ripening cultivars [65]. It has been confirmed that an increased level of patulin in apples positively correlates to the decreased firmness of fruits [66]. Beyond firmness, traits like fruit size, skin color, peel thickness, and cuticular wax, also act as implicit factors affecting the tolerance level of the cultivar to the invading pathogen and patulin production [64,67]. Mainly inherited ancestrally, these physical traits are also affected by sun exposure and the ethylene level, which has a strong connection with the surrounding environmental conditions and chemical constitution. 

The apple fruit contains a wide range of chemicals, which significantly affect host resistance to fungal diseases. The most important chemical constituents that are associated with patulin accumulation in apples are the levels of ethylene, polyphenols, and sugars. 

Known as a major hormone in regulating fruit ripening and softening, ethylene also plays a role in the apple’s defense against pathogen infection. A lower ethylene production rate has been recognized as the major reason of a greater resistance to blue mold decay in late-ripening cultivars [68]. Moreover, the endogenous production of ethylene can be used as an indicator of wound healing in apples [69]. During *P. expansum* invasion, the ethylene production and ethylene-mediated wound response mechanisms were suppressed in apples, which in turn made pathogen colonization much more easier [70]. Therefore, faster wound healing results in less decay and less accumulation of patulin in apples.

Similarly, many abundant polyphenols in apples are also involved in the response to patulin attack through scavenging the free radicals induced by patulin. A study of 24 apple cultivars revealed that flavonols and procyanidins B2 from apple peel and flesh played a significant role in blue mold resistance [71]. Higher concentration of epi-catechin and procyanidin B1 were also found in resistant *M. sieversii* PI 369855, in comparison to susceptible “Golden Delicious” (*M. domestica*) [57]. Both observations supported the possibilities of phenolic compounds to positively contribute—at the basal level—to the resistance of *P. expansum* attacks in apples. 

Another key component of apple fruit that modulates the accumulation of patulin is sugars, particularly sucrose. The addition of glucose, sucrose, or maltose at a final concentration of 10 g/L (about 29.2 mM) to a basal medium (without a carbon source), led to the highest yields of patulin by *P. expansum* strains T01, M1, and Pe21. [54]. However, a decrease of patulin production by *P. expansum* isolates T01 and Pe21 was observed in a solid secondary medium, where the sucrose concentration ranged from 15 to 175 mM [41,72]. The differences in the amount of sucrose within these two culture media could be the reason behind the variations in patulin accumulations. As sucrose makes up the large part of sugars in apple fruits, the changes of sucrose content during fruit ripening may be associated with fungal metabolism and mycotoxin synthesis. 

#### 2.1.3. Environmental Conditions

During apple planting, factors like annual precipitations can directly affect apple yield and quality, from aroma and flavor to flesh firmness, even disease resistance. Heavy precipitations in Hungry and Queretaro led to severe blue mold decays in apples during 2010 and 2011, respectively [64,73]. However, these seasonal factors are unpredictable. Fresh apples continue respiring even after they are harvested. Generally, harvested apples are stored either in air (10–25 °C) for 3–4 months, or in cold, hypoxic conditions (0–4 °C, 1–3% O_2_, and 0.03–5.00% CO_2_) for up to 12 months [74,75]. Environmental changes such as temperature, gas composition, and pH, could affect patulin accumulation by regulating the traits of the apple and the physiological properties of *P. expansum*, like the ability to produce patulin. 

The temperature at storage facilities has an effect on the development of blue mold decay, which directly associates with patulin production in rotten apples. More importantly, the patulin from these rotten apples is likely to pass to the final products if processing fails to remove the mycotoxin. 

Ambient temperature has a crucial effect on the growth and colonial morphology of *P. expansum*, thus the production of secondary metabolites such as patulin in apples could also be affected [76]. Although *P. expansum* can grow well at 0 °C or below, a faster sporulation and mycelia growth have been observed when the storage temperature set at 20–25 °C [52,53,77,78]. The effect of storage temperature on patulin production is more complicated. The highest amount of patulin was found in “Red Delicious”, “Golden Supreme”, “Gala”, “Fuji”, “Empire”, and “McIntosh”, as well as in apple cider samples, when the storage temperature set above 20 °C [50,79]. However, higher levels of patulin were also been observed when “Red Chief” apples and apple sauce were stored at 1 °C or 4 °C instead of 22 °C or 23 °C [80]. *P. expansum* isolates exhibited varying abilities of patulin production when incubated at 1 °C or 20 °C, suggesting a strain specificity in stress conditions [81]. Considering the enormous diversity of *P. expansum* isolates and apple cultivars, it is necessary to investigate in the future how the interactions of pathogen strains and apple cultivars can affect patulin production. 

In juice manufacturing, apples are first sent to pre-treatment, like washing and selection; then pressing and juice holding; followed by juice filtration; and finally, sterilization and bottling [39]. Pasteurization, evaporation, and distillation are the common preservation methods, which are expected to remove the detrimental microorganisms and even mycotoxins. However, the effect of heat treatments on patulin is still controversial, probably due to the differences in the initial concentration of patulin in apple products [40]. A significant reduction of patulin from apple products was only observed in early attempts. Approximately 90% of the original 100–150 µg/mL patulin was removed after 29 h heating at 105 °C [82]. Considering the nutrient loss and the low organoleptic properties after a high temperature treatment for a prolonged time, a high-temperature short-time sterilization is more widespread for processed apple products. However, limited effectiveness has been reported with regard to patulin removal. When apple juice was pasteurized for 30 s at 90 °C, the level of patulin was reduced from 433 µg/L to 262 µg/L, which was a 39.6% reduction [83]. In another trial of apple puree, a 20.7% decrease of the initial 140 μg/L patulin were monitored when the products were heated at 90 °C for 10 min or 30 min, respectively [84]. As patulin is thermally stable in an acidic condition, the natural pH of apple juice could help stabilize the patulin and reduce the effectiveness of any heat treatment.

The composition of gas, particularly O_2_ and CO_2_ in the storage room, has crucial influence on apple ripening and patulin production by *P. expansum*. In general, apples are usually picked during the pre-climacteric stage (unripening) or climacteric stage (commercial ripe), and are sent to storage rooms with a controlled atmosphere, which could help slow down the apple ripening and prolong the shelf-life to supply fresh fruit throughout the year [85]. Not only related to apple ripening, the adjustment of gas composition also affects the flesh firmness of the apple, which plays a role in response to pathogen attack and patulin production. However, the growth of *P. expansum* is not affected by lower O_2_ or higher CO_2_ [32], and the effect of limited oxygen on patulin accumulation in apples is debatable. In a model of apple puree agar media, the effect of reducing the O_2_ level from 20% to 3% on patulin production by *P. expansum* was strain-dependent; while a consistent suppression of patulin production was observed in all of the tested *P. expansum* strains when the O_2_ was dropped from 3% to 1% [86]. In different stages of apple storage, no significant variation of the patulin content was observed, although the content of oxygen changed significantly [44]. Similarly, limited O_2_ storage failed to reduce patulin in the “Galaxy” and “Fuji Kiku” apples from Brazil [46]. These results indicated that the effect of a controlled atmosphere on patulin reduction is a complicated process, which is not only affected by apple cultivars and *P. expansum* strains, but is also affected by abiotic stresses like the temperature of the storage room, the material of the apple package, and the pre-treatment of apples before the storage. 

Changes in the environment pH have less to do with the traits of the harvested apples, but more to do with the physiology and pathogenicity of *P. expansum*, which further determine the production of patulin in apples. Patulin accumulation as well as the spore germination and biomass formation of *P. expansum* can benefit from a lower pH condition (pH 3–5) in apples [54,87]. During *P. expansum* infection, two pH-modulators, d-gluconic acid and ammonia, are secreted and act as the precursors for patulin biosynthesis process [41]. The former acidifies the apple tissues and enhances the colonization of *P. expansum* by activating the cell wall degrading enzyme polygalacturonases [88]. With the sequential consumption of sugars and d-gluconic acid as the carbon source for pathogen growth, ammonium accumulates in apples and then contributes to patulin production by inducing the expression of a pH modulator, PacC [41]. 

### 2.2. Challenges in the Control of Patulin Levels in Apple Products

Patulin accumulation in apple fruits is a complicated process that is deeply affected by the abovementioned factors, like *Penicillium*, apple fruits, and environment. The cross-linked interactions between such factors block the development of the effective control measures of blue mold decay, hence, patulin production in apples. Moreover, the control of patulin levels in food is also challenged by the difficulties in the rapid detection of patulin residues in food matrixes. 

Among all of the developed strategies to reduce blue mold decay and detoxify patulin in apple products, antagonist microorganisms showed superior advantages were hence recognized as promising biological control agents in the fruit industry. However, their safe usage is under scrutiny because of the unspecific degrading mechanism(s), as well as the incomplete toxicological assessment of degradation by-products or derivatives. 

#### 2.2.1. Difficulties of Patulin Detection in Apple Products

Reliable and sensitive assays for detecting patulin within different food matrixes is the primary requirement in managing patulin contaminations. However, the complicated composition of apple products could cause a deviation in the identification and quantification of patulin by compounds that share a similar chromatographic behavior to patulin.

For many years, the most widely used strategy to detect patulin was solvent extraction, followed by a clean-up step, and finally, chromatographic analysis using a reverse phase HPLC coupled with ultra-violet (HPLC-UV) detection at 277 nm [89]. Although HPLC-UV shows good repeatability and high quantitative precision, this method is undermined because of the presence of interfering substances from apple or apple by-products formed during processing [90,91]. Among them, 5-hydroxymethylfurfural (5-HMF), pectin, and some proteins have been found in liquid and dried apple products that interfere patulin determination [18,92]. During heat pasteurization, 5-HMF is formed as a dehydration product of fructose in acidic conditions [93]. As both 5-HMF and patulin have a maximum UV absorption at 276 nm, the appearance of 5-HMF could affect the accuracy of quantifying patulin in foodstuffs [25]. Likewise, pectin with its high abundance is also regarded as an interferer when the extraction and chromatographic separate of patulin from apple products is conducted [94]. Although the inclusion of an enzymatic hydrolysis step is a commonly practiced depectinization approach, such a treatment can result in a significant under estimation of patulin and lower recovery rates of patulin due to the formed pectin hydrolysate [95]. Moreover, an interaction of patulin and approximately 20% proteins occurred in cloudy apple juice, where bound patulin was formed and was difficult to extract using organic solvents [96]. 

Although liquid chromatography-mass spectrometry (LC-MS) has powerful capabilities in separation and identification, this method is challenged by the physicochemical properties of patulin. As a hydrophilic and high polar polyketide, patulin has a 154.0266 Da monoisotopic molecular mass, which is consistent with 13 fungal secondary metabolites, including intermediates from patulin biosynthesis pathways like neopatulin (or isopatulin) [37]. Sharing the same functional groups, neopatulin is an optically inactive isomer, which is difficult to be distinguished by MS [97]. The poor ionization of patulin, not matter in electrospray or in atmospheric pressure chemical ionization conditions, makes it frequently omitted when monitoring multi-mycotoxin in foodstuffs [98]. The high expense of instrumentation is probably another impediment that hinders the promotion of the LC-MS-based detection of patulin in apple products. 

#### 2.2.2. Insufficient Toxicological Assessments of Patulin and Its Breakdown Products

As the only regulated small toxic lactone, the permitted level of patulin in apple-based foods for children, infants, and babies is lower than that for adults [37]. Young children are at greater risk of intoxication because of their specific dietary pattern, low body weight, higher metabolic rate, and poor ability to detoxify hazardous xenobiotics [99]. Therefore, toxic assessments of patulin and its related metabolites should be studied comprehensively. Up until recently, the toxic effects of patulin have been explored in vivo [100], ex vivo [101], and in vitro [102] (Table 2). Long-term exposure to patulin is accompanied with genotoxicity, cytotoxicity, immunotoxicity, neurotoxicity, and severe damage to mammalian organs, especially kidneys and livers [103,104]. Although the long term exposure of rats and mice indicated the potential carcinogenicity of patulin, the International agency for Research on Cancer (IARC) classified patulin in group 3, “not classifiable as a carcinogen to humans” because of insufficient evidence [104].

Compared to patulin, the toxicology of patulin-related metabolites is less investigated, which include degradation products (mainly produced by biocontrol microorganisms) (Table 2) and conjugates (mainly produced by patulin and thiol-contained compounds). Three patulin degradation products have been widely reported, which are desoxypatulinic acid and two isomers of ascladiol (*E*- and *Z*-ascladiol) [89,109]. With opened pyran rings, these breakdown products showed less cytotoxicity to human cell lines than patulin [105,107], but no further reports on their carcinogenicity, genotoxicity, or other toxic effects. The newly found hydroascladiol was produced after the four-week incubation of the first degradation product ascladiol with *Lactobacillus plantarum* [108], for which the toxicity is still largely unknown. Patulin conjugates are produced because of its electrophilic attack to sulfhydryl groups of nucleophiles, such as cysteine and methionine, from live cells [110,111]. The cysteine-containing tripeptide glutathione (GSH; γ-Glu-Cys-Gly) could also become the target to patulin [112]. Although patulin/cysteine conjugates are considered less or nontoxic to intestinal cell lines, the toxicological profiles of 22 (or more) mass spectra identified patulin-GSH adducts remain elusive [112,113,114]. As GSH is a scavenger of free radical and the cofactor for various antioxidant enzymes, its role in patulin detoxification needs further investigation.

#### 2.2.3. Unspecific Mechanisms of Patulin Degradation by Microorganisms

In recent years, the use of antagonistic microbial agents to control postharvest blue mold decay and reduce patulin accumulation in apple products has shown great potential [36]. Some protein (e.g., phosphomannomutase) and polysaccharides from the yeast cell wall could bind patulin [115,116]. As patulin causes the generation of reactive oxygen species (ROS), it is highly possible that antioxidant genes/proteins are responsible for patulin detoxification in cells. However, few reports have disclosed the degrading genes/enzymes involved in the biodegradation pathways. A DNA microarray assisted transcriptome analysis found that the genes involved in the proteasome, sulfur amino acid metabolism, and oxidative stress of *Saccharomyces cerevisiae* were highly induced by patulin [117]. The deletion of superoxide dismutase encoding gene *SOD1* elevated yeast susceptibility to patulin attack [118]. In *Schizosaccharomyces pombe*, the expression of the Cu/Zn superoxide dismutase, catalase, and glutathione S-transferase were increased as a consequence of the activated transcription factor Pap1 by patulin [119]. Moreover, a collection of 3000 T-DNA inserts helped to identify four homologous genes of *S. cerevisiae, YCK2*, *PAC2*, *DAL5*, and *VPS8*, in *Sporobolomyces* sp. IAM13481 were sensitive to patulin treatment [120]. A more recent proteomic profiling revealed that both the abundance of protein U7 (a short-chain dehydrogenase) and the expression of its coding gene were significantly increased by patulin treatment, which suggested the possibility of this protein’s involvement in patulin degradation in *Candida guilliermondii* [121]. As none of these genes have their function validated in vitro, more work needs to be done to prove that patulin degradation is performed through specific enzymes/pathways. 

## 3. Strategies for the Mitigation of Patulin Contamination in Fresh Apples and Processed Products

**Figure 1:**
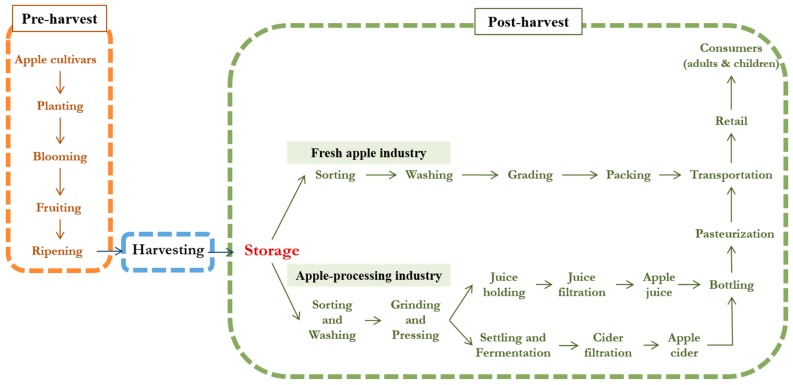
Flowchart of apple from orchard to processing.

During the past few decades, researchers have been dedicated to exploring strategies to reduce patulin contamination in apple products. The Ontario Ministry of Agriculture, Food, and Rural Affairs (OMAFRA) summarized the indicators of higher level of patulin, including the fallen apples, visibly rotten or bruised apples, and apples from atmospherically stored conditions [122]. The most common and effective methods to control patulin level are also listed, which consist of the regular inspection and removal of decayed apple prior to processing. However, the culling of rotten apples will not be as efficient, unless the decayed areas are on the surface and visible. Other strategies are also developed aiming at the control of apple blue mold. Among which, the application of fungicides (e.g., thiabendazole) had already been used to reduce disease incidents [123]. Fungicides with potential risks of chemical residue carryovers and the development of fungicide-resistant species have caused worry among the public, and therefore, have been gradually banned in most countries [124,125]. Considering the fate of apples from orchard to table (Figure 1), green and effective strategies that inhibit apple blue mold and control patulin levels in apple-based products will be discussed in this section. 

### 3.1. Preharvest Control of Blue Mold to Reduce Patulin Accumulation in Apples

Slow-healing wounds and poor resistance against *P. expansum* are two major reasons for patulin accumulation in apples. To achieve the goal of having less patulin in apples and apple products, the preharvest prevention should focus on increasing disease resistance in apples, and the reduction of blue mold incidence and severity in apples, via lowering the spore population and growth rate of *P. expansum* (Table 3).

#### 3.1.1. Selection of Blue Mold Resistant Apple Cultivars

During recent years, a growing number of studies have sought apple cultivars with superior resistance to *P. expansum*. This strategy aims to reduce the decay and accumulation of patulin in apples, even the damaged ones selected from blue mold-resistant apple cultivars. Wild apples with a naturally strong resistance to disease have been considered as promising and sustainable sources in resistant apple breeding, of which *M. sieversii* is the most prominent. With the identification of the qM-*Pe*3.1 QTL among resistant *M. sieversii*, we hope to use these disease resistance alleles in introgression breeding or to act as the molecular markers that help with accelerating the screening of a resistant apple progeny [60]. The value of resistant apple cultivars has also been realized by scientists from Sweden and Norway, who selected 12 apple cultivars with a high resistance to *P. expansum* and an adaptability to cold weather [124]. Further investigations will be needed to breed new apple cultivars by exploring new fungal virulence genes and pathways, which regulate *P. expansum* virulence and patulin production. To meet consumers’ needs, the organoleptic qualities of new apple cultivars should also be taken into consideration, because domesticated apple varieties are significantly bigger, firmer, and sweeter than *M. sieversii* from Kazakhstan [145].

#### 3.1.2. Good Management Practices in Apple Orchards

Good agricultural practices and orchard-management are important in enhancing the storage quality of apples from planting to harvesting, and contribute further to lowering the levels of rotten apples and patulin contaminations.

In orchards, dispersed spores may randomly land on a fruit’s surface by wind or agricultural activities like mowing. Meanwhile, practices such as pruning, mulching, thinning, and fertilizing have varying effects on the prevalence of *Penicillium* rot in apples [146]. Evidence shows that summer pruning (in July and August), bark mulching, and hand thinning positively improved the productivity and/or storability of early season “Katja” and several other organically grown apple cultivars (e.g., “Dayton”, “Delorina”, “Santana”, “Sultanat”, and “Zarya Alatau” in Sweden) [124,147]. Mulching practices, especially cornstalk treatment, also significantly improved the firmness of apples from Loess Plateau (China), which may be beneficial in reducing disease incidence and patulin contaminations [148]. 

During apple harvesting and handling, precautions should be taken to minimize apple bruising and wounding [123]. The selection of an optimal harvest date is also important, as the occurrence of severe rot is more likely to happen in late-harvested apples than in earlier ones [149]. To predict an optimal picking date for various cultivars, the Streif index is widely used as an indicator that relies on firmness, soluble solids content, and starch value [65]. In terms of recommended values, this index is lower in unripe fruit and higher in over-ripe fruit. The “Aroma” apple, which, when picked two weeks after normal harvest time, showed higher incidents of blue mold [149]. Another feasible way to determine the optimal harvest dates is by calculating the days after full bloom (DAFB). However, a lower decay index was observed in both early- or late-ripening apple cultivars, which were harvested at higher DAFB [124]. Therefore, the combination of the Streif index and DAFB in harvest dates determination could extend the shelf-life of apples and, more importantly, result in less decay and patulin accumulation. 

After harvest, fresh apples are usually sent to the warehouse for long-term storage. A hot water washing step before the subsequent storage seems very promising in reducing mold infection and patulin concentrations [130]. Heat shock proteins induced by such a heat treatment could further prepare apples for pathogen attack [128]. Therefore, implementing proper management in orchards and procedures after harvest will contribute to a better control of the *P. expansum* spread, lowering the level of patulin contaminations within the final products.

#### 3.1.3. Preharvest Application of Plant Elicitors

Plant elicitors, if within the right dosage, can elevate the expression of defense-related genes and increase activities of resistance-associated enzymes in fruits [150]. In that case, preharvest treatment with a small number of elicitors could effectively trigger apple resistance to *P. expansum*, and thus, reduce patulin production in apples. In a recent study, the spraying of a 20 mg/L harpin solution on trees 4–8 days prior to the harvest helped in reducing the *P. expansum* infection from 32% to 5–10% and from 30% to 4% in “Empire” and “Red Delicious” apples, respectively [126]. Blue mold decay on “Golden Delicious” apples was significantly reduced after applying 1 mM of ammonium molybdate in some orchards [127]. The effectiveness of these elicitors on inducing apples’ resistance is largely dependent on the type, dosage, *P. expansum* population, and the timing of treatment (before or after harvest) [130,135,151]. Taking harpin as an example, 40 mg/L harpin was required after harvest to achieve equivalent effects of 20 mg/L applied before harvest [126]. Before the large-scale usage of these elicitors, more investigation on the dosage, application time(s), and safety evaluations are needed.

### 3.2. Postharvest Control of Patulin Contamination in Apples and Apple-Products

Once decayed apples enter juice-manufacturing process, it is hard to remove the patulin from the final products. Green and effective methods are needed to meet consumers’ demand for safe and additive-free food preparations. From apple storage to processing, a series of non-thermal and natural detoxification or degradation strategies have been developed to efficiently reduce or remove patulin from apples or apple-products (Table 3).

#### 3.2.1. Control of Patulin Production during Apple Storage

Given the fact that contaminations with *P. expansum* are unavoidable, and that they are responsible for blue mold decay in apples, it is of great importance to prevent mold growth and mycotoxin production during storage. To lower the incidence of apple blue mold, the hygiene of warehouses is of utmost importance. The chances of infection and/or the colonization of *P. expansum* in injured apples are greatly reduced in storage rooms with good sanitary conditions [32]. Moreover, temperature, atmospheric composition, and moisture levels are also considered as critical components to the proper storage of apples. Low temperatures have been widely used in commercial apple storage, for their suppression of *P. expansum* growth [152]. Recently, controlled atmospheres (1.00–3.00% O_2_ and 0.03–5.00% CO_2_) and/or ethylene content adjustments through the spraying of 1-methylcyclopropene have been coupled with cold storage practices to better extend the shelf-lives of apples and to inhibit *P. expansum* spreading [46,153,154]. Although patulin is commonly found during a later storage phase, a high density of *P. expansum* spores could result in a large amount of patulin accumulating in apples, even at an earlier onset of cold storage [44,155]. Therefore, a new trend of applying biocontrol agents (i.e., antagonistic yeast cultures) in cold and hypoxic warehouses has been tested, showing their ability to synergistically inhibit *P. expansum* growth and patulin accumulation [156,157]. As a critical control point in the commercial production of apples, the improvement of storage conditions will bring a multiplier effect in controlling apple blue mold decay and patulin accumulation within processed apple products.

#### 3.2.2. Mitigation of Patulin Contamination during Apple Processing

After a certain period of storage, apples are supplied to grocery stores or are processed into apple products. Treatments like the culling or sorting damaged fruits and the hot water washing/dipping apple fruits ahead of further pressing are proven to be useful and feasible in reducing patulin contamination. The patulin content in unfermented apple cider made from unculled apples could reach 59.9 µg/L–120.5 µg/L, but only reach 0–15.1 µg/L in the cider from culled ones [158]. In a recent study and after an ambient storage for 21–93 days, the level of patulin in “McIntosh”, “Gala”, “Fuji”, and “Golden Supreme” varied from 5920 µg/kg to 54,221 µg/kg [79], which could benefit from a carefully established culling and prevent patulin levels from exceeding the regulatory upper limits within the final products. By sorting out apples with moldy spots over 10 cm^2^, another study showed that the contents of patulin was reduced from 15.8 µg/kg to 1.1 µg/kg and from 8.3 µg/kg to 0.6 µg/kg in cloudy and clear apple juice, respectively [155]. Water treatment on apples also showed a reduction of patulin in the final products. During the processing of apple juice concentrate, the washing stage contributed to a 33.6% reduction of patulin and, thus, is considered as the most effective procedure when compared to an enzymatic treatment (9.9% reduction) or micro-filtration (3.7% reduction) [12]. In apple puree production, a pulping step (i.e., A step to remove peel and cores from apple mash by a pulper with 4 mm and 1.25 mm sieves) before pasteurization also led to an 80% degradation of patulin [84]. The disappearance of patulin has been observed after the alcoholic fermentation of apple juice [159]. The abovementioned procedures in apple processing, except alcoholic fermentation, are practical and economic ways that are expected to reduce patulin contamination in processed apple products intended for adult or child consumption alike. 

#### 3.2.3. Reduction of Patulin Contamination from Apple Products

Ideally, detoxification procedures should not only reduce the content of toxins to safe levels, but also prevent any reduction of the nutritional and palatable values of the treated commodity. In the past, strategies that were applied to reduce patulin contamination in apple products were either physical or chemical. The trend in patulin detoxification has evolved from traditional methods to environmental friendly non-thermal treatments, natural extracts, and biological control agents and their combinations, in order to exert additive even synergistic effects. 

Novel physical technologies have gained attention in the mitigation of patulin from processed apple products, including the application of irradiation, high hydrostatic pressure, and adsorbents [160]. Approximately 90% of 1000 µg/L patulin was removed from extreme UVC (ultraviolet light with wavelength at 222 nm) treated apple juice and cider [139]. Unfortunately, a supplementation is required to remedy the loss of ascorbic acid and other photosensitive substances after the UV treatment. To keep most of the color and natural flavor of liquid apple products, high hydrostatic pressure and ozone detoxification techniques are also applied in patulin detoxification. In mixed apple juice, 30% of the patulin was eliminated in a high pressure treatment at 600 MPa for 300 s [138]. However, the effect of high hydrostatic pressure on patulin degradation highly relies on thiol concentration of juices [40]. More than 90% patulin was removed from the juice through the physical adsorption with chitosan-coated Fe_3_O_4_ particles or water-insoluble corn flour [161,162]. Moreover, the patulin was reduced from 198.36 µg/L to 48.92 µg/L in concentrated apple juice when a self-developed gaseous ozone equipment was set at 12 mg/L ozone purging for 15 min ozonation [144]. The above data collectively shows that non-thermal treatments are indeed promising approaches for controlling patulin contamination if the diminishment of nutritional compounds and the possibility of patulin desorption could be addressed in the near future. 

During the development of alternatives to fungicides, green and natural compounds have grabbed researchers’ attention. Among them, natural antioxidants, plant extracts, and essential oils, showed the ability to suppress the growth of *P. expansum* or the synthesis of patulin. In apple juice that was inoculated with *P. expansum*, the addition of 2 mg/L of Turkish propolis (a natural honeybee hive product) inhibited patulin synthesis from 30.47 µg/L in the first day of storage to 27.63 µg/L after 48 h (*p* < 0.001); while the patulin content in the control was 26.06 µg/L and 56.40 µg/L, respectively [143]. Similarly, the application of a 0.01% (*w*/*v*) bamboo leaf flavonoid inhibited blue mold decay and patulin accumulation, with 0.029 µg/kg in untreated apples and 0.002 µg/kg in treated apples after 20 days of storage at 20 °C [132]. In the presence of 10% of the supernatant of Kombucha (a fermented drink made from black tea, sucrose, acetic acid bacteria, and yeast), a 49.8% inhibition of patulin production has been observed in *P. expansum* inoculated apples [163]. A micro-emulsified essential oil prepared from the leaves of betel vine (≥0.5 µL/mL) also showed promising antifungal effects, especially for the inhibition of mycelial growth of *P. expansum* in apple juice [164]. Antioxidants like ascorbic acid could accelerate the degradation of patulin in apple juice as well [165]. As these bioactive compounds have long been used as food additives or food preservatives, it is hopeful to exploit their abilities against blue mold decay and patulin accumulation within the food industries. 

Microorganisms have been always considered as promising candidates in reducing the exposure to mycotoxins from infected food crops and fruits. Some biocontrol agents are able to adsorb mycotoxins from an aqueous solution, and others can even transform te mycotoxin into less toxic or non-toxic products [166]. Antagonistic yeasts are the most popular biological tools against the blue mold decay of apples in cold or deck storage (Table 4). Known as brewer’s yeast, *Saccharomyces cerevisiae* commonly biodegrades patulin to *E*-ascladiol via the alcoholic fermentation of apple juice to hard cider [159]. Furthermore, a spray developed from a culture filtrate of *S. cerevisiae* contributed to a 48% reduction of blue mold decay and a 42.6% reduction of patulin accumulation in “Golden Delicious” apples [167]. Similarly, the decay incidence was dropped from 65.6% to 35.6% when stored apples were treated with *Pichia caribbica* [168]. As effective as fungicides (imazalil and pyrimethanil), *Metschnikowia fructicola* inhibited *P. expansum* when apples were stored at 1 °C for 56 days [80]. Apples that were treated with *Rhodosporidium paludigenum* also showed a 67% and 100% reduction in the decay incidence and patulin concentration, respectively [131]. Moreover, viable bacteria could biodegrade the patulin effectively. Up to 96% of the patulin was degraded by *Gluconobacter oxydans* in apple juices [169]. *Pseudomonas fluorescens* strain 2–28 was able to reduce the blue mold incidence by 88% in “Spartan” apples, which was comparable to fungicide Scholar^®^ or the registered decay biocontrol agent, Bio-Save^®^ [170]. The adsorption of patulin from an aqueous solution has been found in the existence of inactivated *Saccharomyces* or lactic acid bacterial cells [115,171]. The biocontrol effect of an atoxigenic fungus against patulin-producing fungus has also been investigated most recently. Living cells of atoxigenic *Aspergillus flavus* HF-B1 were capable of reducing 89.5% or 62% of the patulin produced by the toxigenic *Aspergillus terreus* HPA1 in PDA media or in Egyptian apples, respectively [172]. Enzymes are also recognized as promising biocontrol agents in patulin detoxification. More than 70% of the patulin was removed when the apple juice was treated with 0.03 g/mL calcium carbonated immobilized porcine pancreatic lipase at 40 °C for 72 h [141].

In order to explore safe and cost-effective substitutes for fungicides, physical practices or chemical treatments have been integrated with biological control agents to exert an additive effect against postharvest diseases in fruits and their products. A recent review by Zhang et al. has summarized the combined application of some of the physical practices and microbial antagonists against patulin [160], among which there is a strong synergistic effect of *Cryptococcus laurentii* (1 × 10^8^ cells/mL) and chitosan (0.1%, viscosity 12 cP), leading to an 86% reduction in blue mold decay within wounded apples [176]. Similarly, the culture filtrate of the edible mushroom *Lentinula edodes* greatly improved the biocontrol activity of *C. laurentii* against *P. expansum* (a reported enhancement from 85% to 100%), coupled with an increased patulin reduction capacity (from 77% to 99%), respectively [133]. Moreover, *Rhodotorula mucilaginosa* at 1 × 10^8^ cells/mL combined with phytic acid at 4 µmol/mL created an additive effect on the reduction of the patulin content in apples [177]. In a similar fashion, *Pichia caribbica* (1 × 10^8^ cells/mL) and bamboo leaf flavonoids (0.01% *w*/*v*) together significantly inhibited the *P. expansum* growth and reduced the patulin accumulation [132]. In essence, the exploration of integrated strategies could be a promising method against postharvest blue mold rot and patulin accumulation in apples. 

## 4. Conclusions and Future Prospect

Apple fruits and apple products, particularly apple juice, play a major role in human exposure to patulin. The in-depth investigations of the factors and challenges associated with blue mold decay in apples and patulin accumulation in apple-products are essential in order to develop methods that can effectively reduce or remove patulin from the human dietary route. Modulated by environment conditions, interactions between pathogenicity of *Penicillium* species and susceptibility of apple cultivars lead to differences in the severity of blue mold decay and levels of patulin accumulation in apples. The high density of a toxigenic pathogen like *P. expansum* in storage rooms is an indispensable element for patulin production in apples. Moreover, the existence of atoxigenic pathogens could also accelerate the development of blue mold decay. Although injured or bruised apples can hardly avoid pathogen attack, apples with one or more characters like fast wound healing or defense mechanisms, such as relatively firm fruits, lower ethylene production levels, and abundant polyphenols, are less likely to accumulate patulin. The physiochemical characteristics of patulin make it easy for this toxin to survive cold, hypoxic, acidic, or high-temperature conditions. In addition, these properties coupled with the complexity food matrixes, interfere the accurate detection of patulin residues in apple products. To mitigate the patulin content in apple-products, newly developed strategies that are safer and more effective compared with the use of synthetic fungicides are needed. Generally, strategies intended to mitigate patulin contamination from preharvest to postharvest mainly focus on improving the disease resistance of apples, or reducing the patulin content indirectly by controlling apple blue mold, or directly by degrading the accumulated toxin. However, it takes years or up to decades to breed new apple cultivars, which have combined resistant abilities to blue mold, as well as the superior quality traits (e.g., bigger size and/or sweet flavor). The resistance of apples can also be improved by the application of biological elicitors with properly calculated/adjusted dosages. Good agricultural practices and orchard-management could also help maintain the storage quality of apples, so that they have a limited effect on disease development. Except for the basic setup of low temperature and controlled atmospheres, the application of ethylene inhibitors or biocontrol agents on apples during storage could additively control patulin accumulation by suppressing *P. expansum* growth. Practices like culling rotten apples and/or washing with hot water prior to juice pressing are simple yet effective means that are highly recommended. UV and high hydrostatic pressure processing are non-thermal treatments for patulin reduction and have showed limited yet promising successes, at least within the laboratory settings. However, the loss of nutrients and the high cost of the needed instruments could jeopardize their large-scale industrial applications. The addition of green antifungal reagents, such as plant extracts or essential oils, could help reduce the patulin content in apple juice. The commercial application of these natural chemicals in juice production pipelines is obstructed by the economic costs of extraction and concentration steps. By far, the most promising method in patulin reduction is the use of biological control agents that consist of antagonistic yeasts, bacteria, and atoxigenic fungi. Some of these agents can completely and efficiently degrade patulin in vivo or in vitro. However, the toxicity of patulin-degradation by-products and the mechanism(s) behind cellular patulin detoxifications are still not clear and should be intensively investigated. Given the substrate speciality of detoxifying enzymes, patulin-degrading enzyme(s) from antagonistic strains may have great potential for patulin removal from apple products. Collectively, efforts are still needed to make children and adults feel safe while enjoying the health benefits of their desirable processed apple-based products, without concerns with regard to patulin contamination.

## Figures and Tables

**Table 1:** Occurrence of patulin contamination in apples and apple-based products reported in recent ten years (2008–2018).

Type of Product	Country	Year of Samples	Number of Samples (Positive/Total Samples)	Range (µg/kg or µg/L)	Percentage of Samples over 50 µg/L Patulin (10 µg/L for Children’s Food)	Reference
Apple	Pakistan (Punjab)	2017	27/36	<LOD–630.8	55.6%	[17]
Apple juice (including organic and conventional apple juice and juice concentrate)	China (Changchun)	2009	ND/35	<1.2–94.7	20%	[12]
	China (Shaanxi)	2008–2010	568/574	2.5–22.7	0	[13]
	China (Hangzhou)	2015	ND/4	<LOD–16.8	0	[18]
	Portugal (Lisbon)	2007–2009	28/68 ^a^	<LOD–42	0	[19]
	Spain (Navarra)	ND	25/100	<LOD–118.70	11%	[20]
	Spain (Catalonia)	2010–2011	21/47 ^a^	<LOD–36.5	0	[21]
	Serbia (Novi Sad)	2013–2015	54/73	<LOD–65.4	1.4%	[22]
	Argentina	2005–2013	1866/4634	<LOD–19,622	0.8%	[23]
	Pakistan (Punjab)	2017	15/29	<LOD–120.5	6.90%	[17]
	Tunisia	2011	11/30	0–167	ND	[24]
	Malaysia	2012–2013	1/13	<LOD–26.9	0	[25]
Apple jam/marmalade	China (Hangzhou)	2015	ND/4	<LOD–11.0	0	[18]
	Argentina	ND	6/26	17–39	ND	[26]
Apple puree/apple pulp	Argentina	ND	4/8	22–221	ND	[26]
	Spain (Catalonia)	2010–2011	6/46 ^a^	<LOD–50.3	2.1%	[21]
	China (Changchun)	2009	ND/30	<1.2–67.3	36.7%	[12]
Products for babies (including apple juice, apple sauce, and compotes)	Italy	2008–2009	22/60	3–9	0	[27]
	Italy (Campania)	ND	0/26	0	0	[28]
	Tunisia	2011	7/25	0–165	28%	[24]
	Portugal (Lisbon)	2007–2009	5/76	<LOD–5.7	0	[19]
	Spain	2008	0/17	0	0	[29]

ND, not disclosed; LOD, limit of quantification; ^a^ infant drinks are included.

**Table 2:** Structure and producer of patulin and degradation products.

Compound	Structure	Formula	Molecular Weight (g/mol)	Toxicity	Reference
Patulin	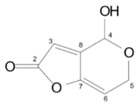	C_7_H_6_O_4_	152.14	Cytotoxic, teratogenicity, mutagenicity, carcinogenicity, developmental and reproductive toxicity, and immunotoxicity	[104,105]
*E*-ascladiol	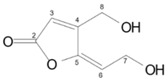	C_7_H_8_O_4_	156.14	30 µM, no cytotoxic to Caco-2	[105]
*Z*-ascladiol	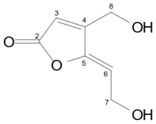	C_7_H_8_O_4_	156.14	30 µM, slight cytotoxic to Caco-2 (20% decrease in cellular proliferation)	[105]
Desoxypatulinic acid	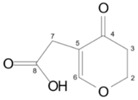	C_7_H_8_O_4_	156.14	50 and 100 µM, slight cytotoxic to human hepatocytes LO2	[106,107]
Hydroascladiol	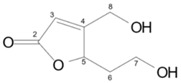	C_7_H_10_O_4_	ND	ND	[108]

ND, not disclosed.

**Table 3:** Newly developed strategies for blue mold control and patulin mitigation in apples and apple products.

Category	Method	Description	Type of Apple Products	*P. expansum* Spore Suspension (spores/mL)	Blue Mold Decay Incidence		Patulin Content		Reference
					Control	Treatment	Control	Treatment	
Orchard	Spray elicitor	Harpin (80 mg/L)	McIntosh apple	5 × 10^3^	70%	30%	ND	ND	[126]
			Empire apple		32%	5–10%	ND	ND	
			Red Delicious apple		30%	4%	ND	ND	
		Ammonium molybdate (1 mM)	Golden Delicious apple	1 × 10^4^	49%	35%	ND	ND	[127]
Before *P. expansum* inoculation	Heat treatment	Hot water dipping (45 °C, 10 min)	Ultima Gala apple	1 × 10^4^	>80%	0%	ND	ND	[128]
		*P. expansum* inoculation, then hot water dipping (2% acetic acid, 50 °C, 3 min)	Red Delicious apple	1 × 10^5^	73.8%	2.2%	ND	ND	[129]
	Biocontrol agents	*Metschnikowia pulcherrima* BIO126 (10^8^ cell/mL)	Golden Delicious apple	1 × 10^5^	100%	56.6%	ND	ND	[130]
		*M. pulcherrima* BIO126 (10^8^ cell/mL) with 10% ethanol			100%	52.3%	ND	ND	
		*M. pulcherrima* BIO126 (10^8^ cell/mL) with 3.0% sodium bicarbonate			100%	56.2%	ND	ND	
		*Rhodosporidium paludigenum* (10^7^ cell/mL)	Fuji apple	1 × 10^5^	100%	67%	0.001 mg/kg	0.03 mg/kg	[131]
		*Pichia caribbica* (1 × 10^8^ cells/mL)	Fuji apple	1 × 10^5^	ND	ND	29 mg/kg	2 mg/kg	[132]
		*Cryptococcus laurentii* LS28 (1 × 10^6^ cells/mL) and *Lentinula edodes* LF23 (2% *w*/*v*)	Golden delicious apple	1 × 10^4^	100%	0	0.47 mg/kg	0.005 mg/kg	[133]
		*Metschnikowia fructicola* AL27 (1 × 10^8^ cells/mL)	Golden delicious apple	1 × 10^5^	ND	ND	>1 mg/kg	0 mg/kg	[80]
	Natural chemicals	Quercetin or umbelliferone (100 μg)	Golden Delicious apple	5 × 10^4^	100%	8% or 14%	65 mg/kg	42 mg/kg or 40 mg/kg	[134]
		Bamboo leaf flavonoid (0.01% *w*/*v*)	Fuji apple	1 × 10^5^	ND	ND	29 mg/kg	2 mg/kg	[132]
	Spray elicitor	β-Aminobutyric acid (50 mM)	Golden Delicious apple	1 × 10^4^	100%	36.6%	ND	ND	[135]
		Ammonium molybdate (5 mM)	Golden Delicious apple	1 × 10^4^	88%	9%	ND	ND	[127]
		*M. pulcherrima* BIO126 (10^8^ cell/mL) and acibenzolar-S-methyl (1 mg/mL)	Golden Delicious apple	1 × 10^5^	100%	57.4%	ND	ND	[130]
After *P. expansum* inoculation	Heat treatment	Hot water dipping (45 °C, 10 min)	Ultima Gala apple	1 × 10^4^	90%	60%	ND	ND	[128]
	Non-thermal processing	Pulsed light (35.8 J/cm^2^, 30 s)	Apple juice	ND	ND	ND	129 mg/L	22.38 mg/L	[136]
		Pulsed light (11.9 J/cm^2^, 20 s)	Apple puree	ND	ND	ND	90 mg/kg	<LOD	
		High hydrostatic pressure (400 MPa, 30 °C, 5 min)	Apple juice	ND	ND	ND	0.05 mg/L	0.024 mg/L	[137]
		High hydrostatic pressure (600 MPa, 5 min)	Apple and spinach juice	ND	ND	ND	0.2 mg/L	0.157 mg/L	[138]
		UV (253.7 nm, 3.00 mW/cm^2^, 40 min)	Apple cider	ND	ND	ND	1 mg/L	0.125 mg/L	[139]
			Apple juice without ascorbic acid	ND	ND	ND	1 mg/L	0.052 mg/L	
			Apple juice with ascorbic acid	ND	ND	ND	1 mg/L	0.014 mg/L	
	Adsorption	Cross-linked xanthated chitosan resin (pH 4, 30 °C, 18 h, 0.01 g)	Apple juice	ND	ND	ND	300 mg/L	170 mg/L	[140]
		Calcium carbonate immobilized porcine pancreatic lipase (40 °C, 18 h, 0.03 g/mL)	Apple juice	ND	ND	ND	1 mg/L	<0.3 mg/L	[141]
		Caustic treated waste cider yeast biomass	Apple juice	ND	ND	ND	0.1 mg/L	0.04 mg/L	[142]
	Natural chemicals	Propolis (2 mg/mL)	Fresh pressed apple juice	0.4 × 10^4^~5 × 10^4^	ND	ND	0.056 mg/L	0.028 mg/L	[143]
		Gaseous ozone (12 mg/L, 10 min)	Apple juice	ND	ND	ND	0.247 mg/L	0.018 mg/L	[144]

ND, not disclosed; LOD, limit of detection.

**Table 4:** Effect of biocontrol agents on patulin degradation.

Strain Type	Strain Name	Strain No. and Source	Active Component and Efficacy	Degrading Condition	Degradation Product	Degradation Mechanism	Reference
Yeast	*Saccharomyces cerevisiae*	S288C; obtained from ATCC (ATCC no. 204508)	Fermentation broth, 3.8 mg/L to 0 mg/L	3.8 ppm PAT; Static Culture, 30 °C, 110 h	*E*-ascladiol and *Z*-ascladiol	ND	[89]
	*Pichia caribbica*	Unsprayed orchard beside Yangtze River, China	Cell-free filtrate, 20 mg/L to 0 mg/L	NYDA/NYDB, 190 rpm, 28 °C, 48 h,	ND	Intracellular and extracellular enzymes	[116]
	*Candida guilliermondii*	Strain 2.63; Obtained from Institute of Microbiology (Chinese Academy of Science)	Live cells, 50 mg/L to 4 mg/L	NYDB media, 28 °C, 200 rpm, 36 h	*E*-ascladiol	A short-chain dehydrogenase (GI: 190348612)	[121]
	*Rhodosporidium kratochvilovae*	LS11; Isolated from olive tree (Italy)	Live cells, 150 mg/L to 3.7 mg/L	LiBa media, 23 °C, 150 rpm, 72 h	DPA	ND	[106]
	*Rhodosporidium paludigenum*	No. 394084; Isolated from South China Sea, preserved by CABI (the U.K.)	Intracellular enzyme, 10 mg/L to 0 mg/L	NYDB media, 28 °C 150 rpm, 48 h	DPA	Biological degradation and physical adsorption	[131]
	*Cryptococcus laurentii*	LS28; Isolated from “Annurca” apples in Molise (Italy)	Live cells, 0.41 mg/kg to 0.08 mg/kg	LiBa media, 25 °C, shaking, 144 h	ND	ND	[133]
	*Sporobolomyces* sp.	IAM 13481; obtained from FGSC (USA)	Live cells, 100 mg/L to 0 mg/L	LiBa media, 24 °C, shaking, 240 h	DPA, *E*-ascladiol and *Z*-ascladiol	Biological degradation, enzymatic reaction	[120]
	*Metschnikowia fructicola*	AL27; Isolated from “Golden Delicious” apples (Italy)	Live cells, 56.4 mg/kg to 0 mg/kg	YEMS media, 22 °C, 100 rpm, 168 h	ND	Competition for nutrients	[80]
	*Kodameae ohmeri*	HYJM25; Isolated from seawater and guts of marine animals	Live cells, 10 mg/L to 0.4 mg/L	YEPD media (pH 4), 28 °C, 100 rpm, 24 h	*E*-ascladiol and *Z*-ascladiol	Might be enzymatic reaction	[173]
Bacteria	*Gluconobacter oxydans*	M8; Isolated from apples with blue-spot in Bari (Italy)	Live cells, 10 mg/L to 0.39 mg/L	PDB media, 30 °C, 175 rpm, 72 h	*E*-ascladiol and *Z*-ascladiol	ND	[169]
	*Bacillus subtilis*	No. 10034; Obtained from CICC (China)	Live cells, 5 mg/L to 0.15 mg/L	Nutrient broth, 25 °C, 150 rpm, dark, 24 h	ND	ND	[174]
	*Rhodobacter sphaeroides*	No. 1.2182; Obtained from CGMCC (China)	Live cells, 5 mg/L to 1.93 mg/L	Seed broth, 25 °C, 150 rpm, dark, 24 h	ND	ND	[174]
	*Agrobacterium tumefaciens*	No. 1.2554; Obtained from CGMCC (China)	Live cells, 5 mg/L to 3.45 mg/L	Seed broth, 25 °C, 150 rpm, dark, 24 h	ND	ND	[174]
Probiotics	*Lactobacillus plantarum*	S1; isolated from fermented animal feeds	Cell free supernatant, 100 mg/L to 0 mg/L	MRS broth, 37 °C, 4 h	*E*-ascladiol, *Z*-ascladiol, and hydroascladiol	ND	[108]
	*Lactobacillus brevis*	LB-20023; Obtained from CICC (China)	Heat-inactivated cells, 4 mg/L to 1.4 mg/L	MRS media, 37 °C, 150 rpm, 48 h	ND	Binding by polysaccharides and proteins from cell wall	[171]
	*Enterococcus faecium*	EF031; Obtained from Aroma-Prox (Cedex, France)	Live cells, 1 mg/L to 0.547 mg/L	BHI broth (pH 4), 37 °C, soft agitation, 48 h	ND	Binding	[175]
Fungus	*Aspergillus flavus*	HF-B1; Isolated from fruits in Egypt	Live cells, 324 mg/L to 34 mg/L	PDB medium, 30 °C, 240 h	ND	ND	[172]

ND, not disclosed; DPA, desoxypatulinic acid; CABI, Centre for Agricultural Bioscience International (the United Kingdom); FGSC, Fungal Genetics Stock Centre, University of Missouri (USA); CICC: China Center of Industrial Culture Collection; CGMCC, China General Microbiological Culture Collection Center.
